# Priming with Recombinant BCG Expressing HTI Enhances the Magnitude and Breadth of the T-Cell Immune Responses Elicited by MVA.HTI in BALB/c Mice

**DOI:** 10.3390/vaccines8040678

**Published:** 2020-11-13

**Authors:** Narcís Saubi, Athina Kilpeläinen, Yoshiki Eto, Chun-Wei Chen, Àlex Olvera, Tomáš Hanke, Christian Brander, Joan Joseph-Munné

**Affiliations:** 1 Vall d’Hebron Research Institute, 08035 Barcelona, Spain; narcis.saubi@vhir.org (N.S.); akilpelainen@irsicaixa.es (A.K.); yoshiki.eto@vhir.org (Y.E.); chun.chen@vhir.org (C.-W.C.); 2 EAVI2020 European AIDS Vaccine Initiative H2020 Research Programme, London SW7 2BU, UK; 3 Irsicaixa AIDS Research Institute, 08916 Badalona, Spain; aolvera@irsicaixa.es (À.O.); cbrander@irsicaixa.es (C.B.); 4 Biosciences Department, Universitat de Vic-Universitat Central de Catalunya (UVic-UCC), 08500 Vic, Barcelona, Spain; 5 The Jenner Institute, Nuffield Department of Medicine, University of Oxford, Oxford OX1 2JD, UK; tomas.hanke@ndm.ox.ac.uk; 6 International Research Center of Medical Sciences (IRCMS), Kumamoto University, Kumamoto 860-8555, Japan; 7 ICREA, Pg. Lluís Companys 23, 08010 Barcelona, Spain; 8 AELIX Therapeutics, 08028 Barcelona, Spain; 9 Microbiology Department, Vall d’Hebron University Hospital, 08035 Barcelona, Spain

**Keywords:** BCG, HIV-1, vaccine, rBCG, HTI, T-cell

## Abstract

The use of *Mycobacterium bovis* bacillus Calmette–Guérin (BCG) as a live vaccine vehicle is a promising approach for HIV-1-specific T-cell induction. In this study, we used recombinant BCG expressing HIVACAT T-cell immunogen (HTI), BCG.HTI^2auxo.int^. BALB/c mice immunization with BCG.HTI^2auxo.int^ prime and MVA.HTI boost was safe and induced HIV-1-specific T-cell responses. Two weeks after boost, T-cell responses were assessed by IFN-γ ELISpot. The highest total magnitude of IFN-γ spot-forming cells (SFC)/10^6^ splenocytes was observed in BCG.HTI^2auxo.int^ primed mice compared to mice receiving MVA.HTI alone or mice primed with BCGwt, although the differences between the vaccination regimens only reached trends. In order to evaluate the differences in the breadth of the T-cell immune responses, we examined the number of reactive peptide pools per mouse. Interestingly, both BCG.HTI^2auxo.int^ and BCGwt primed mice recognized an average of four peptide pools per mouse. However, the variation was higher in BCG.HTI^2auxo.int^ primed mice with one mouse recognizing 11 peptide pools and three mice recognizing few or no peptide pools. The recognition profile appeared to be more spread out for BCG.HTI^2auxo.int^ primed mice and mice only receiving MVA.HTI. Here, we describe a useful vaccine platform for priming protective responses against HIV-1/TB and other prevalent infectious diseases.

## 1. Introduction

After nearly 30 years since the first diagnosed AIDS case, we have been unable to prevent the approximate 1.7 million new infections occurring in 2019 [1]. Despite all the information, education and prevention resources invested in HIV prevention, the number of new infections has only decreased by 40% since the peak in 1997. The prognosis for HIV infected individuals has improved since the onset of the pandemic. Antiretroviral treatment has transformed HIV infection from a deadly infection to a chronic disease for most infected people, but not for all of them. In 2018, only 53% of people living with HIV had achieved viral suppression thanks to proper antiretroviral treatment [2]. With these figures in mind, the development of an effective and affordable HIV preventive vaccine is still an urgent need.

There is strong evidence in favor of a role for HIV-1 specific T-cell responses in the control of HIV-1 replication [3,4]. One promising approach for T-cell induction is *Mycobacterium bovis* bacillus Calmette–Guérin (BCG) as a bacterial live recombinant vaccine vehicle. Specific humoral and cellular immune responses against HIV-1 have been detected after immunization of mice with recombinant BCG (rBCG) expressing HIV-1 antigens [5,6,7,8]. Using BCG as a vaccine vector has advantages over other newly developed vectors: (i) large scale production is cheap and optimized, (ii) good thermostability [9], (iii) can be administered to newborns with no effect of maternal antibodies on vaccine efficacy [10,11], and (iv) mycobacterial cell wall composition are potent adjuvants [12,13,14,15]. BCG safety has been demonstrated over the last 100 years, where around 3000 million doses have been administered [16]. Recombinant BCG has been tested in human clinical trials, either as an improved vaccine vector against *Mycobacterium tuberculosis* [17], and as a vaccine vector against respiratory syncytial virus (RSV) [18]. Regarding the administration of BCG vaccine to HIV-1-infected individuals, the suggestion of the World Health Organization is that infants of unknown HIV-1 status, and with no clinical signs related to HIV-1, are to be vaccinated. In addition, BCG vaccination can be administered after starting anti-retroviral therapy (ART) and once the infant is confirmed to be immunologically stable (CD4 > 25%) [19].

In our group we’ve previously developed several rBCG.HIV-1 vaccine candidates with the aim of inducing protective cell-mediated responses. Our platform is based on a heterologous rBCG prime and recombinant modified viral vectors (modified vaccinia Ankara—MVA, chimpanzee adenovirus—ChAd) boost regimen delivering common immunogens [20]. There is evident data and there has been acceptance that for some vaccines, priming with one vaccine vector and boosting with another expressing the same antigen will give an enhanced cellular immune responses. Our group and other many research groups have already demonstrated in BALB/c mice and non-human primates that rBCG:HIV prime enhance the elicited viral boost vector specific-HIV-1 T-cell responses. On the other hand, we have demonstrated that rBCG expressing HIVA immunogen in combination with MVA boost is safe and immunogenic in adult and newborn BALB/c mice. HIVA, is an immunogen derived from consensus gag protein of HIV-1 clade A, prevalent in Central and Eastern Africa, and a string of CD8^+^ T-cell epitopes [20,21,22]. In addition, we have developed an *Escherichia coli*-mycobacterial shuttle plasmid DNA, p2auxo. This expression vector employs an antibiotic resistance-free mechanism for plasmid selection and maintenance based on glycine complementation in *E. coli* and lysine complementation in mycobacteria, either as episomal [23] or integrative expression vector [24]. With the aim of using MTBVAC [25], the only live-attenuated *M. tuberculosis* based vaccine, as a vector for a dual TB and HIV vaccine, we constructed the recombinant MTBVAC.HIVA^2auxo^ strain and demonstrated in BALB/c mice safety and immunogenicity [26].

As part of the EAVI2020 preventive HIV vaccine program, we have generated recombinant BCG.HIV strains expressing two immunogens designed to elicit HIV-1 specific T-cell immunity. We constructed recombinant BCG expressing immunogens HIVconsv1 and HIVconsv2, designed by the University of Oxford [27]. These are the 2nd-generation conserved-region immunogens aiming to induce effective T-cell responses against conserved regions of the HIV-1 proteome, which are common to most global HIV-1 variants and in which mutations often lead to loss of replicative fitness. The 2nd-generation HIVconsvX immunogens utilizes a bi-valent mosaic design to maximize the match of potential 9-mer T-cell epitopes in the vaccine to global variants [28,29]. In addition, we have also constructed recombinant BCG expressing the HIVACAT T-cell Immunogen (HTI). HTI is a rationally designed mosaic sequence of 529 amino acids in length, covering 16 regions in gag, pol, vif and nef genes that are relatively conserved among different HIV-1 strains. These regions include more than 60 CD4+ and CD8+ T cell beneficial epitopes targeted preferentially by T cells of HIV-1-positive patients with low viral load [30]. In previous experiments, we demonstrated in BALB/c mice safety, genetic plasmid DNA stability and immunogenicity of BCG.HTI^2auxo.int^ when boosted with ChAdOx1.HTI [31].

In this study, we have assessed the BCG.HTI^2auxo.int^ in combination with MVA expressing HTI immunogen, tested genetic stability, safety and immunogenicity in a murine model. We demonstrated that the vaccine was stable in vitro for 35 bacterial generations, and when BALB/c mice was immunized with BCG.HTI prime and MVA.HTI boost, was safe and induced HIV-1-specific T-cell responses. Furthermore, we have observed differences in the breadth of the T-cell immune responses. The recognition profile appeared to be more spread out for BCG.HTI^2auxo.int^ primed mice and mice receiving MVA.HTI only. This vaccine candidate might be a useful tool in the development of an effective vaccine platform for priming protective responses against HIV-1/TB and other prevalent pediatric pathogens.

## 2. Materials and Methods

### 2.1. Construction of the BCG.HTI^2auxo.int^ Strain Using an Antibiotic-Free Plasmid Selection System

**Figure 1:**
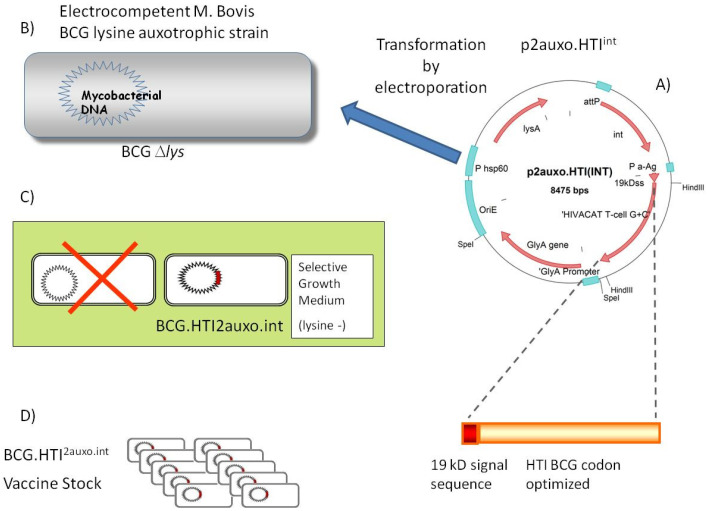
Construction of BCG.HTI^2auxo.int^ vaccine (**A**) The HIVACAT T-cell immunogen (HTI) synthetic DNA coding sequence was *Mycobacterium bovis* bacillus Calmette–Guérin (BCG) codon-optimized and fused to the 19-kDa lipoprotein signal sequence and inserted into the integrative p2auxo.HTI^int^ *E. coli*-mycobacterial shuttle plasmid. This vector contains Pα-Ag, which is a *Mycobacterium tuberculosis* α-antigen promoter, PHSP60, which is a heat shock protein 60 gene promoter. The *glyA* and *lysA* complementing genes function as an antibiotic-free selection and maintenance system in the auxotrophic strains of *E. coli M15*Δ*glyA* and BCGΔ*Lys*, respectively. (**B**) p2auxo.HTI^int^ shuttle plasmid was transformed into lysine auxotroph BCG by electroporation. (**C**) Transformed mycobacteria were selected by plating electroporated cells onto non-lysine supplemented agar medium. (**D**) Colonies were tested for HTI protein expression by Western blot and amplified and stored at −80 °C using the seed-lot system.

Construction and characterization of BCG.HTI^2auxo.int^ has been previously described [31]. The double auxotrophic *E. coli*–mycobacterial shuttle integrative vector expressing the HIVACAT T-cell immungen, p2auxo.HTI^int^ has been previously generated in our laboratory (Figure 1A). Briefly, this vector contains the *E. coli glyA* and mycobacterial *LysA* complementing genes, which function as an antibiotic-free selection and maintenance system in the auxotrophic strains of *E. coli* M15Δ*glyA* and BCGΔ*Lys*, respectively. It also contains sites (*attP*) for integration into the BCG genome at the *attB* site. The synthetic DNA coding sequence of HTI [30], codon-optimized for BCG expression to match the G + C rich mycobacterial codon usage for enhanced expression [32], is fused to the 19-kDa lipoprotein secretion signal sequence, under the control of mycobacterial alpha-antigen promoter. The plasmid DNA was transformed by electroporation into *M. bovis* BCG lysine auxotroph host strain Pasteur Δ*lysA5::res* [33,34]. This BCG parental strain was kindly provided by Jacobs, Bloom, and Hsu. Transformed colonies were selected in non-lysine supplemented agar plates.

### 2.2. Bacterial Cultures

BCG.HTI^2auxo.int^ [31] and BCGwt were cultured in Middlebrook 7H9 broth medium or on Middlebrook agar 7H10 agar medium supplemented with albumin–dextrose–catalase (ADC; Difco Laboratories, Franklin Lakes, NJ, USA) containing 0.05% Tween 80. Cultures were grown at 37 °C in a roller device at 1 rpm up to OD_600_ 1–1.5. Cell cultures were supplemented with sterile glycerol up to 20%, aliquoted and stored at −80 °C.

### 2.3. Viral Strains

MVA.HTI was constructed as previously described [30]. MVA.HTI viral stocks were provided by IrsiCaixa, under material transfer agreement (MTA), and stored at −80 °C until use. MVA.HTI was diluted up to 10^6^ MVA.HTI pfu (plaque forming units)/100 µL with sterile PBS just before mice inoculation.

### 2.4. Immunization of Mice and Isolation of Splenocytes

Groups of eight adult (seven-week-old) female BALB/c mice were immunized intradermally (id) in one footpad, and two groups were left unimmunized. The first group received 10^6^ colony-forming units (CFU) of BCG.HTI^2auxo.int^ (Group A); the second group received 10^6^ CFU of BCG wt (Group B), both groups in one footpad. Two groups were left unimmunized (Groups C and D). Groups A–C were boosted intramuscularly (im) with 10^6^ pfu of MVA.HTI after five weeks, while group D was left unimmunized. All the mice were sacrificed two weeks after the boost for immunogenicity analyses. Immediately following sacrifice of the animals, splenocytes were harvested and homogenized using 70 µm cell strainers (Falcon; Becton Dickinson, Franklin Lakes, NJ, USA) and 5-mL syringe rubber plungers. Red blood cells were removed with ACK lysing buffer (Lonza, Barcelona, Spain), and the splenocytes were washed and resuspended in complete medium (R10 (RPMI 1640 supplemented with 10% fetal calf serum and penicillin–streptomycin), 20 mmol/L of HEPES, and 15 mmol/L of 2-mercaptoethanol).

### 2.5. IFN-γ ELISpot Analysis

The enzyme-linked immune absorbent spot (ELISpot) assay was performed using the commercial murine interferon-γ (IFN-γ) ELISpot kit (Mabtech, Nacka Strand, Sweden) according to the manufacturer’s instructions. The ELISpot plates (MSISP4510, 96-well plates with polyvinylidene difluoride membranes, Millipore, Burlington, MA, USA) were 70% EtOH treated and coated with purified anti-mouse IFN-γ capture monoclonal antibody diluted in phosphate-buffered saline (PBS) to a final concentration of 5 µg/mL at 4 °C overnight. Then, 250,000 fresh splenocytes were added to each well and stimulated with 17 peptide pools containing a total of 14,715 mer overlapping peptides (OLP) spanning the HTI sequence, at a concentration of 10 μg/mL per peptide. To assess HLA-E related responses, splenocytes were stimulated with 4 HLA-E binding peptides derived from the HTI immunogen (RL9-RMYSPTSIL-, SN9-SEELRSLYN-, VI9-VGEIYKRWI- and MD9-MYSPVSILD-) [35,36], along with the leader sequence peptide of HLA-G, (VL9-VMAPRTLFL-) as a control. All the samples and controls were plated in duplicate wells. ELISpot assays were incubated for 16 h at 37 °C, 5% CO_2_. The plates were subsequently washed 5× with PBS, incubated for 2 h with a biotinylated anti-IFN-γ monoclonal antibody (mAb) diluted in PBS 2% Fetal Calf Serum (FCS) to a final concentration of 2 µg/mL, washed 5× in PBS, and incubated with the streptavidin–alkaline phosphatase conjugate in PBS 2% FCS. Then, plates were washed 5× with PBS before incubating with 100 µL of 5-bromo-4-chloro-3-indolyl phosphate (BCIP)/nitro blue tetrazolium (NBT) substrate solution (Sigma-Aldrich, St. Louis, MO, USA). After 5–10 min, the plates were washed with tap water, dried, and the resulting spots counted using an ELISPOT reader (Autoimmune Diagnostika GmbH, Strassberg, Germany). For each animal, the mean of background responses was subtracted individually from all the wells to enable a comparison of the IFN-γ spot forming cells (SFC)/10^6^ between groups. To define positive responses, a threshold was defined as at least five spots per well, and responses exceeding the mean number of spots in negative control wells plus three standard deviations of the negative control wells.

### 2.6. Statistical Analysis

Immunogenicity data is represented as group means of the total IFN-γ SFC/10^6^ response or as medians for individual antigens/pools. Statistical differences were assessed by ordinary one-way analysis of variance when comparing total ELISpot responses or the Kruskal–Wallis test when comparing responses to individual pools. (* *p* < 0.05; ** *p* < 0.01; *** *p* < 0.001). GraphPad Prism 6.0 (Graphpad, San Diego, CA, USA) software was used. Body mass data are shown as group means with error bars indicating standard deviation as well as means ± 2 standard deviations (SD) from naïve mice. Statistical analyses were performed using the Kruskal–Wallis test.

### 2.7. Ethics Statement

The animal experiments strictly conformed to the animal welfare legislation of the Generalitat de Catalunya. All the experiments were approved by the local Research Ethics Committee (Procedure 43.19, Hospital de la Vall d’Hebron, Universitat Autònoma de Barcelona).

## 3. Results

### 3.1. Construction of the BCG.HTI^2auxo.int^ Vaccine Strain

Plasmid p2auxo.HTI^int,^ (Figure 1A), was transformed into electrocompetent lysine auxotrophic BCG strain, Pasteur substrain. (Figure 1B). The positive recombinant BCG.HTI^2auxo.int^ colonies were selected by culturing the rBCG cells on Middlebrook agar 7H10 medium without lysine supplementation (Figure 1C). One clone of BCG.HTI^2auxo.int^ was selected according to HTI protein expression level and was preserved by using the seed-lot system. A Master Seed stock (MS) and derivative Working Vaccine stock (WVS) was prepared (Figure 1D).

### 3.2. The BCG.HTI^2auxo.int^ Prime-MVA.HTI Boost Regimen Elicits HIV-1-Specific T-Cell Responses

**Figure 2:**
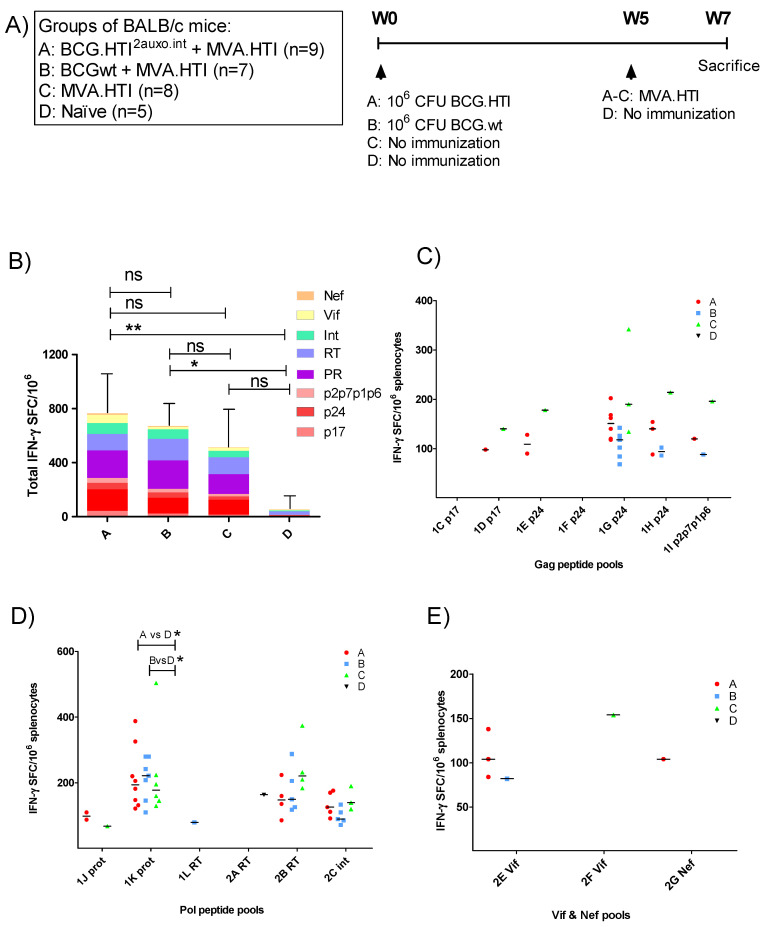
Induction of HIV-1 specific T-cell responses by the BCG.HTI^2auxo.int^ + modified vaccinia Ankara virus HIVACAT T-cell immunogen (MVA.HTI) prime-boost regimen in BALB/c mice. (**A**) Groups and the immunization schedule. Adult mice (seven weeks old, *n* = 5–9/group) were immunized with either 10^6^ cfu of BCG.HTI^2auxo.int^ (intradermally) and boosted with MVA.HTI (10^6^ pfu, intramuscular) after five weeks (group A), or with 10^6^ CFU BCGwt (id) and boosted with MVA.HTI (10^6^ pfu, intramuscular) after five weeks (group B), or only immunized with MVA.HTI (10^6^ pfu, intramuscular) at week five (group C), or left unimmunized (group D). Two weeks post-boost, mice were sacrificed, and splenocytes were isolated for enzyme-linked immune absorbent spot (ELISpot) analysis. (**B**) The total magnitude of HIV-1 specific SFCs/10^6^ splenocytes was calculated as sums of the SFCs elicited by the 17 HTI peptide pools, the color-coding represents the HIV-1 gene location of the pools. Data are presented as group means and error bars represent the standard deviation of the total sum of SFC/10^6^ splenocytes. Statistics were performed using the non-parametric Kruskal–Wallis test adjusted for multiple comparisons, * *p* < 0.05, ** *p* < 0.01 (**C**–**E**). HIV-1-specific T-cell responses interferon-γ (IFN-γ spot-forming cells SFC/10^6^ in response to HTI-derived peptide pools representing HIV-1 gag (**C**), HIV-1 pol (**D**), and nef + vif (**E**). The data are presented as medians of group responses above the threshold. ns: not significant.

We previously demonstrated that priming with BCG.HTI^2auxo.int^ efficiently induced broad HIV-1 specific T-cell responses when combined with ChAdOx1.HTI [31]. While priming with BCGwt also increased the total magnitude of the immune response, these responses were directed toward fewer epitopes. In order to assess the enhancement provided by BCG.HTI^2auxo.int^ in the context of immunization with MVA.HTI, BALB/c mice were immunized as outlined in Figure 2A. The HIV-1 specific T-cell immune responses were evaluated following a short immunization schedule consisting of a BCG.HTI^2auxo.int^ prime and a MVA.HTI boost delivered after 5 weeks. A group primed with BCGwt was included as a control for the unspecific adjuvanticity of BCG. Two weeks after the boost, T-cell responses were assessed by IFN-γ ELISpot upon stimulation with 17 peptide pools spanning the HTI proteome [30]. The highest total magnitude of IFN-γ spot forming cells (SFC)/10^6^ splenocytes was observed in BCG.HTI^2auxo.int^ primed mice compared to mice receiving MVA.HTI alone or mice primed with BCGwt, although the differences between the vaccination regimens only reached trends. The total IFN-γ SFC/10^6^ was significantly different from naïve mice in groups primed with BCG.HTI^2auxo.int^ or BCGwt (Figure 2B). The most reactive peptide pools in all vaccination groups were 1G p24, 1K prot, 2B RT and 2C int, consistent with findings observed when immunizing with ChAdOx1.HTI [31]. However, statistically significant differences were only observed when comparing mice primed with BCG.HTI^2auxo.int^ or BCGwt with non-immunized mice in response to one peptide (Figure 2D; 1K Prot, *p* = 0.0205 and *p* = 0.0108, respectively).

### 3.3. Breadth of T-Cell Immune Responses in BCG.HT^2auxo.int^ + MVA.HTI Immunized BALB/c Mice

**Figure 3:**
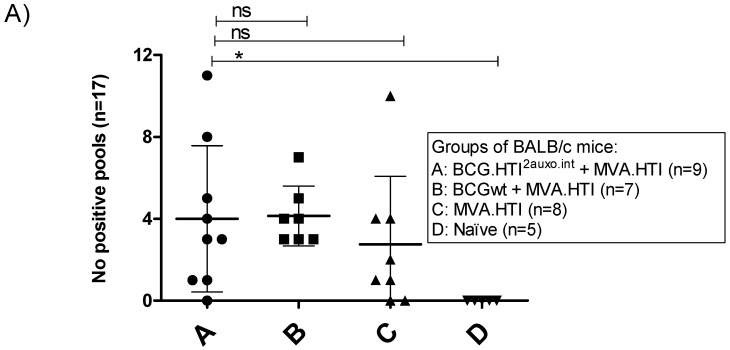

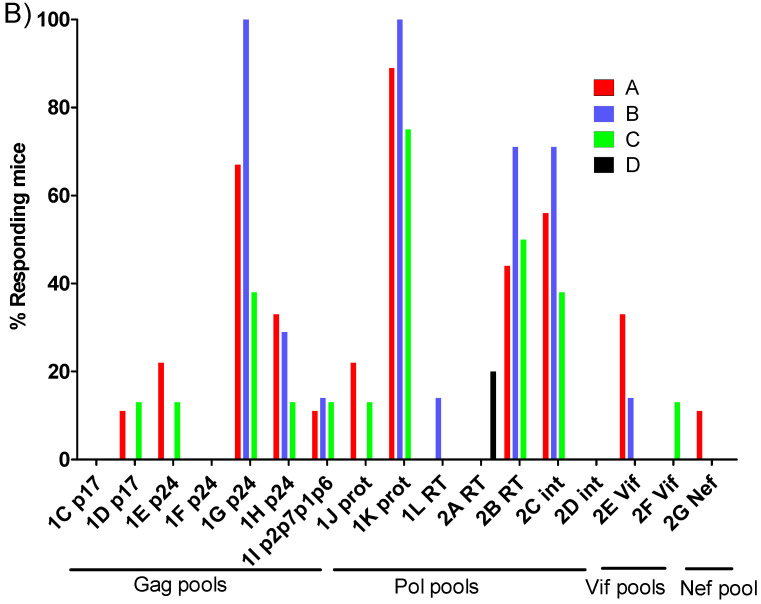
Differential recognition of peptide pools in BCG.HTI^2auxo.int^ + MVA.HTI immunized BALB/c mice. Adult mice (seven weeks old, *n* = 5–9/group) were immunized with either 10^6^ cfu of BCG.HTI^2auxo.int^ (id) and boosted with MVA.HTI (10^6^ pfu, im) after five weeks (group A), or with 10^6^ BCGwt (id) and boosted with MVA.HTI (10^6^ pfu, intramuscular) after five weeks (group B), or only immunized with MVA.HTI (10^6^ pfu, intramuscular) at week five (group C), or left unimmunized (group D). Two weeks post-boost, mice were sacrificed, splenocytes were isolated for IFN-γ ELISPOT analysis, and the numbers of reactive peptide pools (total *n* peptide pools = 17) were compared for each mouse. (**A**) The number of reactive pools per mouse. Statistics were performed using the non-parametric Kruskal–Wallis test adjusted for multiple comparisons, * *p* < 0.05. (**B**) The percentage of reactive mice in each group according to peptide pool and HIV-1 gene location. ns: not significant.

As we previously observed differences in the breadth of the immune response between mice primed with BCG.HTI^2auxo.int^ and BCGwt, we examined the number of reactive peptide pools per mouse. Interestingly, both BCG.HTI^2auxo.int^ and BCGwt primed mice recognized an average of 4 peptide pools per mouse. However, the variation was higher in BCG.HTI^2auxo.int^ primed mice with one mouse recognizing 11 peptide pools and three mice recognizing few or no peptide pools (Figure 3A). On the other hand, when comparing the percentage of responding mice per peptide pool, all BCGwt primed mice respond to the more dominant peptides 1K prot and 2B RT, whereas the recognition profile appeared to be more spread out for BCG.HTI^2auxo.int^ primed mice and mice only receiving MVA.HTI, with these groups having a few mice responding to less recognized peptide pools such as 1D p17, IE p24, 1J prot (Figure 3B). Even though we could not observe statistically significant differences, we could appreciate a high trend in the breath.

BCG vaccination has previously been shown to induce HLA-E restricted T-cell responses [37]. To assess whether priming with recombinant BCG could have an impact on HIV-1 specific HLA-E restricted T-cell responses, we stimulated splenocytes of vaccinated mice with four HLA-E binding peptides derived from the HTI immunogen along with the leader sequence peptide of HLA-G, VMAPRTLFL (VL9) as a control. Mice were immunized similarly to the immunogenicity assay outlined in Figure 2A, but we included one group of animals vaccinated with BCG.HTI^2auxo.int^ not boosted. No statistically significant differences in IFN-γ secretion were observed between mice primed with BCG.HTI^2auxo.int^ or BCGwt, nor in mice immunized with MVA.HTI alone or in those not receiving any immunization. However, mice primed with BCG.HTI^2auxo.int^ and boosted with MVA.HTI displayed significantly higher cumulative HLA-E specific IFN-γ secretion (*p* = 0.0048) and specifically in response to the HLA-E binding peptide (SN9, *p* = 0.0221) when compared with mice vaccinated with BCG.HTI^2auxo.int^ only (data not shown).

### 3.4. The BCG.HTI^2auxo.int^ + MVA.HTI Prime-Boost Regimen Is Safe in BALB/c Mice

**Figure 4:**
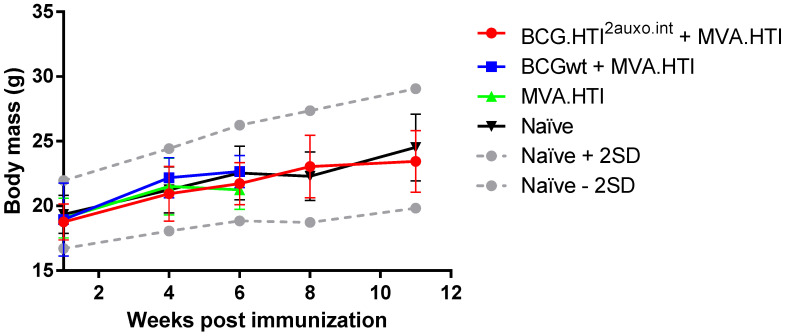
Safety of the BCG.HTI^2auxo.int^ and MVA.HTI prime-boost regimen in BALB/c mice. Mice in groups of five (female, 7-weeks old) were immunized intradermally with 10^6^ CFU of BCG.HTI^2auxo.int^ or BCGwt and boosted with 10^6^ pfu of MVA.HTI (im). Body weights were recorded regularly, and the mean for each group of mice is shown as mean ± SD (*n* = 5). Data from naive mice are presented as mean ± 2 SD (*n* = 5) (dashed grey lines).

Five adult mice per group were either left unimmunized or received 10^6^ colony forming units (cfu) of BCG wt or a total of 10^6^ cfu of BCG.HTI^2auxo.int^ intradermally and 5 weeks later were boosted with 10^6^ pfu of MVA.HTI (im) and their body mass was monitored regularly over time (Figure 4). The body mass profile was similar to the mouse provider’s standard body mass curve (www.envigo.com). Furthermore, no statistically significant difference in body weights was observed between the vaccinated mice and the non-vaccinated mice at any time point tested (Figure 4). Mice were monitored weekly for signs of malaise. No vaccine-related deaths, no local adverse events, and no associated systemic reactions were observed (data not shown).

## 4. Discussion

The development of an effective HIV-1 preventive vaccine includes several important steps. One of them is the design and selection of proper immunogens. Once the immunogens have been designed, the next step is the selection of a suitable vaccine vector to elicit the desired and specific immune responses. As part of the EAVI2020 HIV vaccine program, two immunogens have been designed to elicit specific-HIV-1 T-cell immune responses: (i) the HIVconsvX immunogen [27] and (ii) the HIVACAT T-cell immunogen (HTI) [30]. Our group has been responsible for constructing the recombinant *Mycobacterium bovis* BCG strains expressing these immunogens. We have built-up BCG.HIVconsv1^2auxo.int^, BCG.HIVconsv2^2auxo.int^ and BCG.HTI^2auxo.int^ vaccine strains and these vaccine candidates are going to be administered in combination with recombinant viral vectors expressing the same HIV-1 antigens, the ChAdOX1 and MVA. In this study, we have constructed BCG.HTI^2auxo.int^ vaccine strain expressing the HTI immunogen, designed and developed by Mothe et al. [30]. We have evaluated safety and immunogenicity after BALB/c mice immunization with BCG.HTI^2auxo.int^ prime, in combination with MVA.HTI delivered as a boost. This combination of vaccine vectors has shown excellent results in previous trials [20]. We first constructed BCG.HTI^2auxo^ strain containing the episomal p2auxo *E. coli*-mycobacterial shuttle plasmid [23], but it was not stable in vitro. Genetic rearrangements and disruption of HTI gene expression were detected (data not shown). Thus, the use of the episomal expression vector was not considered. It was previously demonstrated by our group that the in vitro genetic stability of the integrative plasmid p2auxo.int expressing HIVA immunogen was higher than the episomal plasmid in BCG in the absence of selective pressure [24]. Thereby, we engineered the BCG.HTI^2auxo.int^ vaccine strain harbouring the integrative antibiotic-resistance free *E. coli*-mycobacterial shuttle vector, p2auxo.int [22], and it was genetically and phenotypically characterized. The presence of the HTI gene was confirmed and the level of HTI protein expression was detected in vaccine stocks cell lysates. Regarding the induction of HIV-1 specific immune responses, our readout focused on full length HTI, using 17 peptide pools containing a total of 14,715-mer overlapping peptides (OLP) spanning the full HTI immunogen. We employed a short immunization schedule of 7 weeks. A group receiving BCGwt as a prime was included to allow comparison of the unspecific adjuvanticity that BCG immunization confers. We evaluated the total magnitude of HIV-1 specific SFCs/10^6^ splenocytes upon peptide pools stimulation. We also evaluated the number of reactive pools per mice and the percentage of reactive mice in each group according to peptide pool and HIV-1 gene location. When assessing the individual peptide pool responses, the most reactive were peptides 1G p24, 1K prot, 2B RT and 2C int, consistent with observations from previous data where BCG.HTI^2auxo.int^ was combined with ChAdOx1.HTI [31]. These data were compared with other vaccine vectors expressing HTI, such as DNA.HTI developed by Mothe et al. [30], and the integrase defective lentiviral vector vaccine expressing HTI, developed by Gallinaro et al. [38]. In Gallinaro’s paper, they stated that pools 6 and 7, covering HIV protease and reverse transcriptase respectively, were the most reactive in mice experiments. In Mothe’s paper, no individual peptide pool data were provided, but a trend showing gag and pol as main targets to induce specific-HIV-1 T-cell responses was shown [30].

In our study, when MVA.HTI was used as a vaccine vector alone, the percentage of IFN-γ SFC/10^6^ splenocytes elicited by peptide pools, according to the HIV-1 protein, was the following: 32% against gag, 62% against pol, 4% against nef and 1% against vif. On the other hand, when we used ChAdOx1.HTI alone in a previous study, the distribution was different: 7% against gag, 89% against pol, 3% against nef and 1% against vif. However, the average results after mice immunization with DNA.HTI alone, in the study by Mothe et al. [30], were as follows: 42% against gag, 52% against pol, 3% against nef and 3% against vif. The differential recognition of peptide pools from different HIV proteins were similar after MVA.HTI and DNA.HTI immunization and enhanced breath was observed in comparison with ChAdOx1.HTI. Thus, with these results in mind, it seems that using MVA.HTI or DNA.HTI alone, the immune response raised against the HTI immunogen is not focussed on pol peptides only, is more distributed among gag peptides and pol peptides. Interestingly, when BCG.HTI^2auxo.int^ was used as a priming agent in combination with MVA.HTI, similar balanced and broad response to most of the proteins included in the immunogen was observed in all animals primed with MVA.HTI alone. These data is in concordance to what was observed by Mothe et al. after C57BL/6 mice immunization with DNA.HTI and boosted with MVA.HTI [30]. In the assays performed by our group, where BCG.HTI^2auxo.int^ was used as a priming agent, either using ChAdOx1.HTI or MVA.HTI (the present paper) as a boosting agent, slight changes in the distribution of the IFN-γ SFC/10^6^ among the four proteins was observed. Notably, the use of MVA.HTI as compared to ChAdOx1.HTI as a boosting vector increased the relative response to HIV-1 gag [31].

The average number of peptide pools against which the different vaccinated groups were reactive was the same for both, BCG.HTI^2auxo.int^ and BCGwt primed mice (4 pools), and slightly lower on average for mice receiving only MVA.HTI (3.5 pools). However, there was a clear difference in terms of variability of number of reactive pools. In the BCGwt primed group, no animal reacted to less than 3 peptide pools, and no animal reacted to more than 7 pools. Contrarily, in mice receiving MVA.HTI only and mice receiving both BCG.HTI^2auxo.int^ and MVA.HTI, there were mice that did not respond to any pool, and mice that responded up to 10 and 11 pools respectively. A similar effect was observed regarding the breadth of the response when mice were boosted with ChAdOx1.HTI, but the mean number of recognized peptide pools was higher. Specifically, both mice receiving ChAdOx1.HTI only and those receiving BCG.HTI^2auxo.int^ + ChAdOx1.HTI recognized a higher number of peptide pools, and there were no mice failing to respond to any pools [31]. Regarding the number of reactive mice upon stimulation with the assessed peptide pools, the dominant pools are consistent (1G p24, 1K prot, 2B RT, 2C int) regardless of which viral vector is administered as a boost. In the present vaccination schedule, the highest % of responding mice to these peptide pools was observed in group B, BCGwt primed mice, but after vaccination with BCG.HTI^2auxo.int^ + MVA.HTI or MVA.HTI only, the recognition profile appeared to be more spread out. (i.e., 1D p17, 1E p24, 1Jprot, 2F Vif, 2G Nef). This results are not statistically significant, but are consistent with the previously observed behavior of increased breadth when mice were vaccinated with BCG.HTI^2auxo.int^ and boosted with ChAdOx1.HTI [31]. This slightly different behavior of number of responding pools, responding % of mice and HIV protein distribution of spots might suggest that ChAdOx1.HTI and MVA.HTI stimulate different T-cell immune populations, and that it may be worthwhile employing both viral vectors in the same vaccination schedule. A similar approach was assessed by Rosario et al. [39], when rhesus macaques were primed with BCG.HIVA and boosted with either ovine Atadenovirus or MVA, or both expressing HIVA. Using both boosting viral vectors, more robust HIV-1-specific T-cell responses were elicited. Other authors have also shown that mice immunization by using several viral vectors, enhance the specific-HIV-1 T cell immune responses [40,41,42].

Non-classical class Ib MHC-E molecule is becoming an increasingly interesting component of the immune response. It is involved in both the adaptive and innate immune responses to several chronic infections including HIV-1 and tuberculosis [36]. BCG vaccination has previously been shown to induce HLA-E restricted T-cell responses. Joosten et al. [37] described human T-cell responses to *Mtb*-derived peptides containing predicted HLA-E binding motifs and binding-affinity for HLA-E. They observed CD8 + T-cell responses to novel HLA-E binding peptides of *Mtb*, which have cytotoxic as well as immunoregulatory activity. HLA-E restricted responses may be of interest for vaccine development since HLA-E presents limited polymorphism and is resistant to downregulation by nef during HIV infection. To date, few human HLA-E-binding peptides derived from HIV-1 have been reported and the role of HLA-E in regulation of HIV-1 infection remains understudied. We’ve included some of them in the present study to assess HLA-E specific binding to HIV-1 peptides. In this current study, although we observed significantly higher cumulative HLA-E specific IFN-γ secretion, we did not detected an evident impact of BCG on HIV-1 specific HLA-E restricted T-cell reponses after mice immunization with BCG.HTI^2auxo.int^ prime and MVA.HTI boost.

## 5. Conclusions

We could conclude that: (i) The use of BCG.HTI^2auxo.int^ as a priming agent increased the magnitude and the breadth of the T-cell immune responses elicited by MVA.HTI; (ii) when priming with BCGwt, the magnitude of the T-cell responses were lower than BCG.HTI^2auxo.int^; (iii) when BCG.HTI^2auxo.int^ was used as a priming agent and MVA.HTI as a boosting agent, slight changes in the distribution of the peptide pools reactivity among the four HIV proteins were observed; (iv) mice vaccination with BCG.HTI^2auxo.int^ in combination with MVA.HTI was safe. We suggest that further studies, assessing the use of more than one viral vector boost after recombinant BCG priming, and a wide range of T-cell immunological tests to evaluate the magnitude, breath and potential HIV restricted HLA-E T-cell immune responses, should be performed.
